# Is a Technically Challenging Procedure More Likely to Fail? A Prospective Single-Center Study on the Short- and Long-Term Outcomes of Inguinal Hernia Repair

**DOI:** 10.1155/2018/7850671

**Published:** 2018-04-01

**Authors:** M. R. Berndsen, Tomas Gudbjartsson, Fritz Hendrik Berndsen

**Affiliations:** ^1^Department of Surgery, Sahlgrenska University Hospital, Gothenburg, Sweden; ^2^Department of Cardiothoracic Surgery, Landspitali University Hospital, Reykjavik, Iceland; ^3^Department of General Surgery, HVE Akranes County Hospital, Akranes, Iceland

## Abstract

**Background and Aims:**

The aim of this prospective single-center study was to evaluate the outcome of inguinal hernia repair.

**Materials and Methods:**

A total of 485 inguinal hernias (452 patients and 33 patients with bilateral hernias) were operated between January 2004 and December 2010. Mean age was 56 years, and 93% were male. Patient demographics and operative data were collected, and the operating surgeon assessed the technical difficulty of the operation. Five years after surgery, a questionnaire evaluated recurrence and chronic discomfort according to the Cunningham scale. 372 responded (82%), and mean follow-up was 5.5 years.

**Results:**

There were 390 repairs for a primary and 62 for a recurrent hernia. Totally extraperitoneal (TEP) operation was most frequently performed (56%), transabdominal preperitoneal (TAPP) operation in 31%, and Lichtenstein and Shouldice in 12% and 2%, respectively. At 5-year follow-up, the primary outcome of chronic discomfort was 19.5%. The independent positive predictors were young age and operation for a recurrent hernia (OR: 3.7), with TEP operation reducing the risk of chronic discomfort (OR: 0.5). The secondary outcome was the recurrence rate of 2.5%. Risk factors were strenuous work (OR: 13.7), technically difficult repairs (OR: 7.2), and chronic discomfort (OR: 6.7).

**Conclusions:**

Every fifth patient had chronic discomfort in long-term follow-up. The recurrence rate was 2.5%, and a technically difficult procedure was a risk factor.

## 1. Introduction

Inguinal hernia repair is among the most common surgical procedures performed worldwide [1], the estimated annual incidence of inguinal hernia repair being 130–160 operations per 100,000 inhabitants [2, 3]. Ninety percent of the patients are males, and the operations are most commonly performed in two age ranges: 1–5 years and 55–80 years [4].

Today, the greatest challenge in inguinal hernia surgery is to avoid recurrences and postoperative chronic groin pain/discomfort [5–8]. After prosthetic meshes were introduced in the early 1980s for hernia repair, recurrence of inguinal hernia after herniorrhaphy has decreased by 50–75% [8, 9]. This is reflected in the Swedish Hernia Registry, where the rate of reoperation due to recurrence of inguinal hernia decreased from 16.4% in 1992 to 8.8% in 2014 [3]. However, numerous studies with thorough long-term follow-up have shown that the rate of chronic postoperative pain is high and that it is one of the major complications affecting patients that undergo hernia repairs [5, 10].

Surgical technique and the level of operative difficulty can be of importance when evaluating long-term results. It has previously been shown that individual surgeon results can vary dramatically and this has been attributed to incorrect surgical technique [11]. However, it has not been assessed if there could be a correlation between the rate of long-term complications and how difficult the repair was technically.

The primary aim of this study was to prospectively assess the short-term and long-term results of inguinal hernia surgery in a cohort of patients in Iceland. The main endpoints were the rates of recurrent hernia and chronic pain/discomfort together with analysis of risk factors, including the operations' level of technical difficulty.

## 2. Materials and Methods

### 2.1. Patients

Demographic and operative data, together with postoperative outcomes of 452 patients with a total of 485 inguinal hernias (33 bilateral hernias), were collected in a prospective database for consecutive patients who were referred to Akranes County Hospital between 1 January 2004 and 31 December 2010. This was a prospective clinical case series study, mainly aimed at quality control. At the time of operation, the operating surgeon registered patient data and intraoperative data. The hospital records were also checked for complications and readmissions.

Information on age, gender, employment status (heavy/light occupational exertion and elderly), and preoperative physical status classification according to the American Society of Anesthesiologists (ASA) was registered. The hernia was classified as primary or recurrent and whether it was right-sided, left-sided, or bilateral. Intraoperative data included the type of hernia (indirect, direct, femoral, or combined) and the type of procedure (TAPP, TEP, Lichtenstein, or Shouldice). Operative time was defined as the time from skin incision to completion of the wound dressing, in minutes. Type of admission (outpatient/inpatient) and hospital stay in days were recorded. All the operations were done by the same surgeon, and at the end of each operation, the same surgeon subjectively classified the operation as having been easy, medium, or difficult, according to how technically challenging it was.

The open procedures were performed either according to the Lichtenstein technique [12] or the Shouldice technique [13]. The laparoscopic procedures were either performed with the transabdominal preperitoneal (TAPP) procedure with titanium staples for mesh fixation [14] or the totally extraperitoneal (TEP) procedure without fixation of the mesh except for bilateral hernias where the mesh was fixated with absorbable PDS tackers [15].

### 2.2. Clinical Follow-Up

All patients were invited to participate in a follow-up programme whereby they would be examined at a 4-week follow-up and then receive a follow-up questionnaire 5 years after the surgery. During the first 5 years of the study, patients received a follow-up questionnaire both at 3 and 5 years after surgery, but then, the study protocol was amended and included only a 5-year follow-up. For the patients that answered both the 3- and 5-year follow-up, the later answer was registered, and if patients only answered the 3-year follow-up that answer was registered.

Seventeen patients were not included in any follow-up, and 63 patients were only included in the short-term follow-up. A total of 372 patients (82%) were included in the long-term follow-up, with a mean follow-up time of 5.5 years (range: 3.2–6.5). Of the 452 patients in the study, 27 (6%) had died at the time of long-term follow-up. In total, 53 patients (12%) were lost to long-term follow-up, 13 of whom had moved abroad (25% of those lost to follow-up).

The primary outcome variables of the study were chronic pain/discomfort (yes/no and classified as mild, moderate, or severe according to the Cunningham scale) at 4-week and 5-year follow-up. Due to the small sample size of recurrences, the recurrence rate was a secondary outcome. If a patient reported having a recurrent hernia in the questionnaire, he/she was contacted and was offered a clinical examination.

### 2.3. Statistics and Approvals

Data were registered in Excel, and data analysis was performed using RStudio and R Statistics 3.2.2 (The R Foundation, Austria). Probabilities (*p* values) of less than 0.05 were considered to be statistically significant.

Descriptive analysis of the data was applied with calculation of mean, median, and percentages. Unadjusted associations between patient characteristics and the primary and secondary outcomes were examined. Fisher's exact test was used for categorical variables, and the Mann–Whitney *U* test or Student's *t*-test was used for continuous variables based on group size and normality of the distribution of the data. The effect of independent variables on chronic pain/discomfort was evaluated using logistic regression analysis.

The study was approved by the Icelandic National Bioethics Committee and the Icelandic Data Protection Commission.

## 3. Results

### 3.1. Demographics and Intraoperative Data

Table 1 shows the demographics and intraoperative data for all patients according to the different operative techniques. The mean age was 55 years, and 418 (93%) of the patients were males. Altogether, 393 patients (87%) were classified as having ASA physical status I-II, 20 patients (4%) as ASA class III, and one patient (0.2%) as ASA class IV. Out of 452 patients, 180 (40%) had occupational exertion that was classified as heavy and 140 patients (31%) as light, and 132 (30%) patients were retired, students, or disabled pensioners.

Unilateral hernia was diagnosed in 419 (93%) of the patients, and most operations were primary repairs (389, 87%). Of the 63 patients who were operated on for recurrent hernia, 50 had had the first recurrence, 11 had the second recurrent hernia, one patient was diagnosed with the third recurrent hernia, and one patient had the fifth recurrence. The most frequent type of hernia was indirect (228, 50%), followed by direct (155, 34%), combined (56, 12%), and femoral (8, 2%).

The most frequent operation technique was laparoscopic, or in 387 of the patients (86%); TEP repair was performed in 249 patients (55%) and TAPP repair in 138 patients (31%). Conventional open hernia repair was performed in 65 patients (14%) and Lichtenstein of which Shouldice operations in 54 patients (12%) and 11 patients (2%), respectively. The mean operative time was 45 min (range: 14–180), 37 minutes for unilateral surgery (range: 14–165) and 57 minutes for bilateral repair (range: 30–180) (*p* < 0.001). The mean operative time was 13 min shorter for the laparoscopic procedure compared to the open group (40 versus 53 min; *p*=0.003).

Three-quarters of the patients (*n*=342, 76%) were operated in an outpatient setting, and the other 110 patients (24%) were admitted to the hospital overnight. The patients who were admitted were significantly older than the patients who were operated in an outpatient setting (mean age 74 versus 49 years, resp.; *p* < 0.001). The mean hospital stay was 2.6 days (median: 2, range: 1–15).

The operations were classified technically by the operating surgeon, whereby 285 (63%) were evaluated as having been easy, 113 (25%) medium difficult, and 53 (12%) difficult.

### 3.2. Short-Term Follow-Up

Out of the 452 patients, 356 (79%) had a follow-up visit 4 weeks postoperatively, and none of them had been diagnosed with a recurrent hernia, but at that time, 24 of the 356 patients (7%) reported having pain in the operative (groin) area. The median duration of absence from work was 10 days for laparoscopic hernia repair (range: 1–30) and 13 days for open surgery (range: 8–21) (*p*=0.048).

Complications at 30 days were diagnosed in 22 patients (4.7%) and were divided into minor and major. The minor complications included 9 haematomas/seromas (1.9%) and five cases of urinary retention (1.1%), with five other patients being diagnosed with superficial wound infection (1.1%). No deep infections (including mesh infections) were diagnosed.

Three patients were diagnosed with a major complication (0.6%): a small bowel injury (0.2%) due to adhesions, acquired at the entry to the abdomen in a TEP operation; a large infected seroma after a TEP operation (0.2%); and intestinal obstruction due to adherence of small bowel between the mesh and the abdominal wall after a TAPP repair (0.2%).

The 30- and 90-day operative mortality was 0%.

### 3.3. Long-Term Follow-Up

A recurrent hernia was found in 10 patients' groins at long-term follow-up, giving a recurrence rate of 2.5%, the rate being 2.3% for primary and 3.6% for recurrent hernia, respectively (*p*=0.64). The mean time from operation to recurrence was 24.3 months (range: 6–43 months). Patients who were operated for recurrent hernia with a laparoscopic (TAPP or TEP) procedure in the primary operation were operated with an open procedure in the repair and vice versa.

The univariate relationship between patient features and the risk of recurrent hernia is shown in Table 2. Patients who had heavy occupational exertion were significantly more likely to have a recurrent hernia (OR: 13.7, 95% CI: 1.9–60.4). There was a significant correlation between a technically difficult operation, graded by the operating surgeon, just after the operation, and recurrent hernia at long-term follow-up (OR: 7.2, 95% CI: 1.6–32.7). Patients who had chronic pain at long-term follow-up were significantly more likely to have recurrent hernia (OR: 6.7, 95% CI: 1.5–33.1).

In total, 78 patients (19.5%) reported having chronic pain/discomfort at long-term follow-up. Of those, 72 patients (18% of all patients) had mild complaints that did not interfere with their daily lives; however, six patients (1.5%) reported having occasional pain/discomfort that did interfere with their daily lives. No patients reported suffering from pain on a daily basis. An analysis of the relationship between patient features and chronic pain/discomfort is shown in Table 3. Patients with chronic pain/discomfort were 5 years younger on average (OR: 0.98, 95% CI: 0.96–0.99; *p*=0.02). Patients who were operated for a recurrent hernia were significantly more likely to have chronic pain/discomfort at follow-up (OR: 3.7, 95% CI: 1.9–7.1; *p* < 0.001). Patients who underwent a TEP operation had significantly less pain in long-term follow-up than patients who underwent other types of operation (OR: 0.5, 95% CI: 0.3–0.9; *p*=0.008).

In the multivariate analysis, young age (OR: 0.98, 95% CI: 0.96–0.99; *p*=0.009) and an operation performed for recurrent hernia (OR: 3.7, 95% CI: 1.9–6.9; *p* < 0.001) were independent positive prognostic factors for chronic pain/discomfort at long-term follow-up.

## 4. Discussion

This study shows a low recurrence rate with a correlation between a recurrent hernia and a technically difficult operation. Furthermore, a significant number of patients reported having discomfort at long-term follow-up, but in most cases, the discomfort was described as minor.

The total recurrence rate in the study was 2.5% in long-term follow-up, 2.3 for a primary repair and 3.6 for recurrent repairs. Due to the small numbers of recurrences, a multivariate analysis of potential risk factors to identify recurrences was not possible. To the best of our knowledge, the evaluation of the technical difficulty of the operation presented here has not been done in any other study on inguinal hernia repair [16]. The single-surgeon design of the study required the operator to subjectively classify the technical difficulty immediately after surgery. Although this evaluation was subjective, a correlation between a technically difficult operation and long-term recurrence rate was found. Similar findings were reported by Kaafarani et al. who found a correlation between the rate of recurrence and the surgeon's level of frustration during 1,622 inguinal hernia repairs (808 open repairs and 813 laparoscopic repairs). There was also a significant correlation between frustration and postoperative complications (OR: 1.27, 95% CI: 1.03–1.56), both for open repair and laparoscopic repair. We agree on the authors' conclusion that it is imperative to optimize the surgical technique and surroundings to minimize the risk of postoperative complications. The present univariate analysis also showed that heavy occupational exertion was a significant risk factor for hernia recurrence. This is most likely explained by the increased intra-abdominal straining with hard labor, as has previously been demonstrated [17].

After the introduction of large randomized studies and registries, it has become evident that chronic pain and discomfort after inguinal hernia surgery is more prominent among patients than previously believed. In the present study, 19.5% of the patients reported having discomfort in long-term follow-up, which is in line with numerous studies―although both higher and lower rates have been reported [18–21]. In this context, it is important to keep in mind that the rate depends on how strict the definition of chronic pain/discomfort is, and this definition can differ between studies [18, 20]. In the present study, the answers were not evaluated independently and every patient who complained of discomfort was registered as having pain/discomfort, irrespective of how severe/mild the symptoms were. Still, most patients (92%) with pain/discomfort in our study described having only mild symptoms that did not interfere with their daily lives.

The univariate analysis showed that a TEP operation was protective against chronic pain/discomfort (OR: 0.5) in long-term follow-up. Several other studies have similar findings, and it can be presumed that the reason could be the absence of mesh fixation in the TEP repair compared to the other repairs [20, 22]. The difference in our study, however, was not statistically significant in the multivariate analysis―most likely due to the small sample size and therefore a lack of statistical power. Young age and repeated hernia repairs, however, turned out to be independent risk factors for chronic pain in the multivariate analysis, which is consistent with the findings of other studies [18, 20, 23].

The operations took 45 minutes on average: 53 minutes for open repair and 40 minutes for laparoscopic repair. Some randomized studies comparing open and laparoscopic repairs have found a 5- to 14-minute longer operation time for laparoscopic repair [24, 25]. This probably reflects the fact that the laparoscopic operations have a longer learning curve as other studies have shown that the operation time for a senior surgeon in laparoscopic hernia surgery is comparable and even shorter than that required for open repair [3, 26].

In the present study, the length of sick leave was shorter for laparoscopic repair than that for open repair, that is, 10 days on average for laparoscopic surgery (range: 1–30) as compared to 13 days for open surgery (range: 8–20). Numerous other studies have shown an absolute difference in return to usual activities in favor of laparoscopic repair [18, 25, 26].

### 4.1. Limitations and Strengths

The main strength of this study was that the follow-up was long (mean: 5.5 years) and the response rate was good (>80%).

Due to the small sample size of recurrent hernias, multivariate analysis of risk factors could not be performed. In many previous studies, a questionnaire combined with selective clinical examination has been used to evaluate the outcome of inguinal hernia surgery [6, 11, 27]. Some reports have found this method to be unreliable although the advantage is the high follow-up rate achieved [28]. Furthermore, it can also be assumed that patients with discomfort would attend a follow-up program regardless of its form.

## 5. Conclusions

This prospective single-center series shows a five-year recurrence rate of only 2.5%. A correlation between a technically difficult operation and recurrence in long-term follow-up was found, but further studies are needed to confirm this correlation. However, every fifth patient complains of chronic groin pain/discomfort at long-term follow-up, although these complaints are minor for most patients.

## Figures and Tables

**Table 1:** Demographics and intraoperative data comparing laparoscopic and open hernia repair.

	Laparoscopic surgery (*n*=387)	Open surgery (*n*=65)	Total (*n*=452)
Mean age (range) (years)	56 (21–95)	53 (18–92)	55 (18–95)
Male, *n* (%)	362 (94)	56 (86)	418 (93)
Heavy occupational exertion, *n* (%)	160 (41)	26 (40)	186 (41)
Mean operative time (range) (min)	40 (14–180)	53 (25–165)	45 (14–180)
Outpatient setting	298 (77)	43 (66)	341 (75)
Unilateral	356 (92)	63 (97)	419 (93)
Primary repair	343 (87)	46 (71)	389 (87)
Hernia type			
Indirect inguinal	180 (46)	48 (74)	228 (50)
Direct	144 (37)	11 (17)	155 (34)
Combined	45 (12)	5 (8)	50 (11)
Femoral	7 (2)	1 (2)	8 (2)
Technical difficulty			
Easy	243 (63)	42 (65)	285 (63)
Medium difficult	101 (26)	12 (19)	113 (25)
Difficult	43 (11)	10 (15)	53 (12)

Numbers of patients are given with percentages in parenthesis, except for age and operation time, where mean with range in parenthesis is given. Laparoscopic surgery: TAPP and TEP; open surgery: Lichtenstein and Shouldice.

**Table 2:** Univariate analysis of the risk factors for a recurrence of hernia following hernia repair.

	No recurrence (*n*=390)	Recurrence (*n*=10)	OR (95% CI)	*p* value
Age (years)	57	48		ns
Operation time (min)	44	63		ns
Heavy occupational exertion, *n* (%)	156 (40)	9 (90)	13.7 (1.9–60.4)	0.002
Recurrent hernia, *n* (%)	54 (14)	2 (20)	1.6 (0.2–8.1)	ns
Technical difficulty, *n* (%)				
Easy/medium	343 (88)	5 (50)		
Difficult	47 (12)	5 (50)	7.2 (1.6–32.7)	0.005
Chronic pain	71 (18)	6 (60)	6.7 (1.5–3 3.1)	0.005

ns: not significant.

**Table 3:** Univariate and multivariate logistic regression analysis of risk factors for chronic pain following inguinal hernia repair.

	No pain	Chronic pain	Univariate analysis		Multivariate analysis	
	(*n*=322)	(*n*=78)	OR (95% CI)	*p* value	OR (95% CI)	*p* value
Age (years)	57	53	0.98 (0.96–0.99)	0.02	0.98 (0.96–0.99)	0.009
Recurrent hernia, *n* (%)	33 (8.5)	23 (25.7)	3.7 (1.9–7.1)	<0.001	3.7 (1.9–6.9)	<0.001
Operation type, *n* (%)						
TEP	184 (57)	32 (41)	0.5 (0.3–0.9)	0.008	0.6 (0.4–1.0)	0.059
Others	138 (43)	46 (59)				

Others: TAPP, Shouldice, and Lichtenstein.

## References

[1] Rutkow I. M. (2003). Demographic and socioeconomic aspects of hernia repair in the United States in 2003. *Surgical Clinics of North America*.

[2] Primatesta P., Goldacre M. J. (1996). Inguinal hernia repair: incidence of elective and emergency surgery, readmission and mortality. *International Journal of Epidemiology*.

[3] Swedish Hernia Register (2018). http://www.svensktbrackregister.se/index.php?lang=en.

[4] Burcharth J., Pedersen M., Bisgaard T., Pedersen C., Rosenberg J. (2013). Nationwide Prevalence of Groin Hernia Repair. *PLoS One*.

[5] McCormack K., Scott N. W., Go P. M., Ross S., Grant A. M. (2003). Laparoscopic techniques versus open techniques for inguinal hernia repair. *Cochrane Database of Systematic Reviews*.

[6] Arvidsson D., Berndsen F. H., Larsson L. G. (2005). Randomized clinical trial comparing 5-year recurrence rate after laparoscopic versus Shouldice repair of primary inguinal hernia. *British Journal of Surgery*.

[7] Bemdsen F., Sevonius D. (2002). Changing the path of inguinal hernia surgery decreased the recurrence rate ten-fold. Report from a county hospital. *European Journal of Surgery*.

[8] Burcharth J., Andresen K., Pommergaard H.-C., Bisgaard T., Rosenberg J. (2014). Recurrence patterns of direct and indirect inguinal hernias in a nationwide population in Denmark. *Surgery*.

[9] Köckerling F., Stechemesser B., Hukauf M., Kuthe A., Schug-Pass C. (2016). TEP versus Lichtenstein: which technique is better for the repair of primary unilateral inguinal hernias in men?. *Surgical Endoscopy*.

[10] Neumayer L., Giobbie-Hurder A., Jonasson O. (2004). Open mesh versus laparoscopic mesh repair of inguinal hernia. *New England Journal of Medicine*.

[11] Eklund A. S., Montgomery A. K., Rasmussen I. C., Sandbue R. P., Bergkvist L. A., Rudberg C. R. (2009). Low recurrence rate after laparoscopic (TEP) and open (Lichtenstein) inguinal hernia repair: a randomized, multicenter trial with 5-year follow-up. *Annals of Surgery*.

[12] Amid P. K., Shulman A. G., Lichtenstein I. L. (1996). Open ‘tension-free’ repair of inguinal hernias: the Lichtenstein technique. *European Journal of Surgery*.

[13] Welsh D. R., Alexander M. A. (1993). The Shouldice repair. *Surgical Clinics of North America*.

[14] Arregui M. E., Navarrete J., Davis C. J., Castro D., Nagan R. F. (1993). Laparoscopic inguinal herniorrhaphy. Techniques and controversies. *Surgical Clinics of North America*.

[15] McKernan J. B., Laws H. L. (1993). Laparoscopic repair of inguinal hernias using a totally extraperitoneal prosthetic approach. *Surgical Endoscopy*.

[16] Sniðmát meistaraverkefnis HÍ - Marta Rós Berndsen.pdf (2017). https://skemman.is/bitstream/1946/26999/1/Marta%20Ro%CC%81s%20Berndsen.pdf.

[17] Flich J., Alfonso J. L., Delgado F., Prado M. J., Cortina P. (1992). Inguinal hernia and certain risk factors. *European Journal of Epidemiology*.

[18] Langeveld H. R., van’t Riet M., Weidema W. F. (2010). Total extraperitoneal inguinal hernia repair compared with Lichtenstein (the LEVEL-Trial): a randomized controlled trial. *Annals of Surgery*.

[19] Alfieri S., Amid P. K., Campanelli G. (2011). International guidelines for prevention and management of post-operative chronic pain following inguinal hernia surgery. *Hernia*.

[20] Eklund A., Montgomery A., Bergkvist L., Rudberg C. (2010). Chronic pain 5 years after randomized comparison of laparoscopic and Lichtenstein inguinal hernia repair. *British Journal of Surgery*.

[21] Gutlic N., Rogmark P., Nordin P., Petersson U., Montgomery A. (2016). Impact of mesh fixation on chronic pain in total extraperitoneal inguinal hernia repair (TEP): a nationwide register-based study. *Annals of Surgery*.

[22] Lau H. (2005). Fibrin sealant versus mechanical stapling for mesh fixation during endoscopic extraperitoneal inguinal hernioplasty: a randomized prospective trial. *Annals of Surgery*.

[23] Koning G. G., Wetterslev J., van Laarhoven C. J. H. M., Keus F. (2013). The totally extraperitoneal method versus Lichtenstein’s technique for inguinal hernia repair: a systematic review with meta-analyses and trial sequential analyses of randomized clinical trials. *PLoS One*.

[24] Grant A. M. (2002). Open mesh versus non-mesh repair of groin hernia: meta-analysis of randomized trials based on individual patient data. *Hernia*.

[25] Berndsen F., Arvidsson D., Enander L. K. (2002). Postoperative convalescence after inguinal hernia surgery: prospective randomized multicenter study of laparoscopic versus Shouldice inguinal hernia repair in 1042 patients. *Hernia*.

[26] Eklund A., Rudberg C., Smedberg S. (2006). Short-term results of a randomized clinical trial comparing Lichtenstein open repair with totally extraperitoneal laparoscopic inguinal hernia repair. *British Journal of Surgery*.

[27] Patel L. Y., Lapin B., Gitelis M. E. (2017). Long-term patterns and predictors of pain following laparoscopic inguinal hernia repair: a patient-centered analysis. *Surgical Endoscopy*.

[28] Vos P. M., Simons M. P., Luitse J. S., van Geldere D., Koelemaij M. J., Obertop H. (2003). Follow-up after inguinal hernia repair. Questionnaire compared with physical examination: a prospective study in 299 patients. *European Journal of Surgery*.

